# Predictions and goals modulate use of partner's gaze during joint action with humanoid robots^☆^

**DOI:** 10.1016/j.actpsy.2026.107838

**Published:** 2026-10

**Authors:** J. Perez-Osorio, E. Wiese, A. Abubshait, A. Wykowska

**Affiliations:** a Technische Universität Berlin, Berlin, Germany; b George Mason University, Fairfax, VA, USA; c Wichita State University, Kansas, USA; d Instituto Italiano di Technologia, Italy

**Keywords:** Gaze cueing, Gaze orienting, Gaze-weighting heuristic, Human-robot interaction, Joint action, Cue reliability, Task difficulty, Second-person neuroscience

## Abstract

During joint actions, partners coordinate attention and actions by using each other's gaze to predict upcoming actions and align with shared goals. Although gaze orienting has been extensively studied using screen-based paradigms, it remains unclear whether partner reliability and action goals calibrate the use of the partner's gaze during embodied interactions. We addressed this using a humanoid robot as a co-actor in two experiments. In Experiment 1, participants performed a color-sorting task while the robot provided informative or noninformative gaze cues. Cue reliability was manipulated between participants: one group experienced a robot whose gaze was informative in 80% of trials, and another at chance level. Task difficulty was manipulated within participants. Only the reliable group made fewer errors on valid than invalid trials under high-difficulty. In Experiment 2, participants completed a handover task in which an auditory cue established an action plan, the robot looked toward a goal-congruent or incongruent object, and a target appeared at a valid or invalid location. Participants responded faster at the cued location only when the robot looked toward the goal-matching object, with eye tracking confirming covert attentional shifts. Together, these findings show that the use of the partner's gaze in embodied joint action operates as a flexible, utility-weighting heuristic. Observers weighted gaze according to its value, upweighting gaze from a reliable partner and when it aligned with shared goals and down-weighting gaze that conflicted with predictions. These results advance understanding of gaze use as a predictive, context-dependent mechanism with implications for reliance on social signals during joint action with humans and artificial agents.

## Highlights

• Use of a partner's gaze in joint action operates as a flexible, utility-weighting heuristic.
• Robot gaze was followed more when the partner was reliable, and the task was difficult.
• Goal-incongruent robot gaze modulated orienting in a live handover task.
• Eye-tracking confirmed effects reflect covert attention, not overt eye movements.
• Findings extend gaze cueing research to embodied joint action settings.

## 1 Introduction

From helping someone reach the salt container on a dinner table to performing a symphony, successful joint action requires coordinating our intentions and actions with others to achieve shared goals (Sebanz et al., 2006; Vesper et al., 2010). A fundamental mechanism supporting such coordination is attention to social signals like gaze direction, which reveals where another's attention is focused and flags potentially relevant objects or locations in the shared environment (Adolphs, 2003; Gallagher & Frith, 2003; Tomasello et al., 2005). During joint action, gaze serves not merely to indicate locations of interest but to establish common ground, signal upcoming actions, and align partners' representations of shared tasks (Sebanz & Knoblich, 2009). A wealth of studies has demonstrated that observers reliably orient their attention in the direction of another's eyes (Friesen & Kingstone, 1998; see McKay et al., 2021, for a recent review). We conceptualize the use of a partner's gaze during joint action as a flexible, utility-weighting heuristic, which we call the gaze-weighting heuristic. On this view, observers weigh the partner's gaze according to its expected utility for the current task, a flexible cognitive policy tuned by context, knowledge, expectations and goals. This heuristic view is particularly relevant in joint action contexts, where partners must continuously calibrate their reliance on each other's signals in response to task demands and past interaction history (Kuhn et al., 2018).

Although gaze orienting was once considered largely reflexive, occurring even when another's gaze is counter-predictive of the target location (Driver et al., 1999; Friesen & Kingstone, 1998), converging evidence shows robust top-down modulation. Beliefs about the gazer's mental states and social relevance, prior experience with cue reliability, and expectations derived from context and task goals all shape whether and how people use the gaze of others (Abubshait et al., 2020; Abubshait & Wiese, 2017; Dalmaso et al., 2020; Gobel et al., 2018; Perez-Osorio, Müller, Wiese and Wykowska, 2015, Perez-Osorio, Müller and Wykowska, 2017, Perez-Osorio, Abubshait and Wykowska, 2021; Wiese, Wykowska, Zwickel and Müller, 2012, Wiese, Zwickel and Müller, 2013, Wiese, Müller and Wykowska, 2014). However, screen-based paradigms typically isolate gaze from the temporal structure of goal-directed action, often using static faces and abrupt shifts from direct to averted gaze (Pfeiffer et al., 2011; Wiese, Metta, & Wykowska, 2017). In everyday interaction, gaze is temporally coupled to action. It forecasts upcoming steps, supports joint coordination, and aligns with shared intentions (Frith & Frith, 2006; Sebanz & Knoblich, 2009; Tomasello & Carpenter, 2007).

This coupling has a mechanistic basis, as eye movements are tightly linked to action planning, with fixations preceding manipulation and previewing task-relevant objects (Flanagan & Johansson, 2003; Hayhoe et al., 2003). Observed actions activate goal and implementation schemas that generate predictions, supported by posterior superior temporal sulcus (pSTS) and related networks sensitive to expectancy violations (Baron-Cohen, 1997; de C. Hamilton & Grafton, 2008; Frith & Frith, 2006; Rizzolatti et al., 2001; Wolpert et al., 2003). Consistent with this account, orienting is larger when gaze fits action expectations and engages both early sensory and later parieto-occipital processes (Perez-Osorio, Müller, Wiese and Wykowska, 2015, Perez-Osorio, Müller and Wykowska, 2017). Despite robust findings from controlled laboratory displays, decisive tests of how these factors jointly calibrate the gaze-weighting heuristic during real, temporally unfolding joint action exchanges remain scarce. We aim at evaluating whether the gaze-weighting heuristic can be selectively deployed in live exchanges, attenuated when it conflicts with task constraints, and amplified when gaze fits ongoing goals and comes from a reliable partner.

### 1.1 Humanoid robots as joint action partners

Humanoid robots afford controlled yet embodied tests by embedding gaze within meaningful action sequences while allowing for precise manipulation of various factors and parameters, such as reliability or temporal characteristics of the gaze behavior (Wykowska, 2021; Wykowska et al., 2016). To test predictions about gaze in joint action under controlled conditions, we employ humanoid robots as co-actors. Using robots as joint action partners enables embodied, temporally-extended interactions while providing experimental control difficult to achieve with human confederates, a unique methodological advantage (Wykowska et al., 2016). Humanoid robots can engage in goal-directed action sequences with pre-programmed reliability and alignment, allowing researchers to systematically manipulate whether a partner's gaze supports or conflicts with shared task goals.

Examining gaze orienting during structured collaborative interactions with humanoid robots provides insights into the flexibility of joint action mechanisms and how humans calibrate their reliance on partners' signals based on situational demands. This approach yields both theoretical advances in understanding how the gaze-weighting heuristic operates during dynamic joint action and behavioral benchmarks that can inform the design of robotic systems capable of natural, adaptive social coordination. This approach aligns with the second-person neuroscience perspective, which emphasizes studying social cognition during online interaction rather than offline, passive observation (Redcay & Schilbach, 2019; Schilbach et al., 2013). Traditional gaze-cueing paradigms present participants with static or pre-recorded stimuli, placing them in a third-person, observational stance. In contrast, interactive paradigms where participants engage in joint tasks with responsive partners, whether human or robotic, engage neural systems and behavioral patterns that are most likely closer to those that are activated in real-life settings (Pfeiffer et al., 2013; Wilms et al., 2010). By embedding gaze within live, goal-directed exchanges where the robot acts as a co-actor in a shared task, we create a second-person context that more closely approximates the demands of real-world joint action.

Recent studies show that humans interpret social signals conveyed by artificial agents, and robots with human-like features (e.g., eyes, facial structure, mouth) trigger attentional shifts comparable to those elicited by human faces (e.g., Chaminade & Okka, 2013; Kompatsiari, Tikhanoff, Ciardo, Metta and Wykowska, 2017, Kompatsiari, Pérez-Osorio, De Tommaso, Metta and Wykowska, 2018, Kompatsiari, Ciardo, Tikhanoff, Metta and Wykowska, 2019; Perez-Osorio et al., 2021; Wiese et al., 2012; Wiese, Shaw, Lofaro and Baldwin, 2017). Importantly, gaze-cueing from robots is modulated by top-down factors, particularly when robots are perceived as socially relevant due to specific visual or behavioral features (Abubshait & Wiese, 2017; Wiese et al., 2012; Wiese, Buzzell, Abubshait and Beatty, 2018; Wykowska et al., 2014). Such effects have been demonstrated not only using static or video stimuli but also in ecologically valid real-time human-robot interactions (Abubshait & Wykowska, 2020; Kompatsiari, Tikhanoff, Ciardo, Metta and Wykowska, 2017, Kompatsiari, Pérez-Osorio, De Tommaso, Metta and Wykowska, 2018; Wiese, Weis, & Lofaro, 2018; Marchesi et al., 2025). Furthermore, robots have been successfully employed to deploy social signals like gaze direction and head orientation in complex contexts. For example, to support storytelling (Mutlu et al., 2006), facilitate collaboration (Boucher et al., 2012), enhance perceived competence (Huang & Thomaz, 2011), provide task-relevant guidance (Mutlu et al., 2009), and improve efficiency in object-sorting tasks (Abubshait et al., 2023; Perez-Osorio et al., 2021).

Neurophysiological evidence reinforces these findings. Shifts in gaze and head orientation by humanoid robots elicit early ERP components (e.g., P1 and N1) that resemble those evoked by human cues, with enhanced amplitudes for valid relative to invalid cues (Wykowska et al., 2014; Kompatsiari et al., 2018). Collectively, these results suggest that people engage similar attentional and cognitive mechanisms when processing social signals from humanoid robots as from humans (Wykowska et al., 2020). Nonetheless, whether this processing stems from perceiving robots as genuine social agents or whether it reflects the engagement of more general mechanisms, such as attentional orienting or causal inference, remains an open question.

Despite the growing potential, relatively few studies have directly linked robot social signals to performance in goal-directed behavior. Abubshait et al. (2024) showed that in a sorting task, participants tended to follow a humanoid robot's gaze and were more accurate when the robot looked toward the goal-congruent bin than when it looked toward the incongruent bin. In that paradigm, the robot handed a colored cube to the participant and then gazed toward either the correct or incorrect bin. Crucially, the task was framed either as cooperative (team scoring) or individual (participants scored only for themselves). This framing modulated gaze following, with the collaborative frame enhancing reliance on the robot's signals.

At the neural level, incongruent gaze elicited greater conflict-related activity, and collaboration was accompanied by larger alpha activity, indicating heightened responsiveness to the robot's social cues. These findings indicate that the deployment of the gaze-weighting heuristic is top-down regulated by whether the interaction is framed as joint action versus individual performance, providing initial evidence that the heuristic is flexibly calibrated based on the relational structure of the task. After the tasks, participants were asked informally about their strategy. They reported that when the task was difficult, they relied on the robot's signal to choose their final answer. However, systematic manipulations of action congruency, task difficulty, and, critically, agent reliability during real-time interaction remain scarce. Addressing these factors with humanoid robots provides an ecologically grounded test of how expectations, goals, and performance history calibrate gaze guidance in interactive contexts. We expect gaze use benefits might be attenuated by the complexity of real-life interactions.

### 1.2 Aim of present studies

Although orienting to others' gaze is robust (Friesen & Kingstone, 1998; McKay et al., 2021), most evidence comes from passive observation of screen-based stimuli that strip gaze from the temporal dynamics of joint goal pursuit (Pfeiffer et al., 2011; Wiese, Metta, & Wykowska, 2017). Furthermore, it is well-established that gaze use is subject to top-down modulation. What remains understudied is which factors influence this modulation when gaze is embedded in real, goal-directed joint action. We propose that task and partner characteristics are crucial. These two interact and allow us to predict and adjust our behavior during joint action. On this basis, we adopt the view that gaze guidance during joint action is a flexible, utility-weighted policy, which we call the gaze-weighting heuristic, tuned by task demands, partner reliability, and shared goals (Kuhn et al., 2018; Wiese et al., 2012; Wykowska et al., 2014). Our experiments address fundamental questions about how context calibrates the gaze-weighting heuristic in joint action: When does gaze guidance support coordination, and when is it attenuated or overridden? Critically, we ask whether these top-down influences, well documented in controlled settings, hold in interactive, temporally unfolding exchanges in which coordination failures have consequences. It remains unclear whether top-down modulation of gaze use reflects a genuine coordination mechanism or a laboratory artifact. If it is a true joint-action mechanism, it should not only survive the transition to embodied interaction but also be sharpened by it, since the value of a reliable partner's signal is highest precisely when miscommunication is costly. Establishing such modulation in embodied settings provides an ecological test of how high-level predictions and partner models shape attention during joint action (Frith & Frith, 2006; Sebanz & Knoblich, 2009; Tomasello & Carpenter, 2007). Humanoid robots enable this test with precise control over when gaze aligns or conflicts with action goals while embedding cues in meaningful sequences (Abubshait et al., 2024; Boucher et al., 2012; Huang & Thomaz, 2011; Mutlu, Forlizzi and Hodgins, 2006, Mutlu, Yamaoka, Kanda, Ishiguro and Hagita, 2009; Perez-Osorio et al., 2021; Wykowska et al., 2016).

Experiment 1 examined how task difficulty and gaze reliability in a goal-oriented sorting task influence how participants use a partner's gaze when making a choice. Experiment 2 examined how goal congruency shapes gaze-cued attentional orienting in an object-handover task. Together, the two experiments probe how top-down modulation acts on the gaze-weighting heuristic at two points in the processing stream: whether a partner's gaze informs the participant's choice (Experiment 1), and whether a partner's gaze draws attention to a location (Experiment 2). This distinction maps onto the three factors we manipulate, which operate at three different levels of the task. Reliability is a partner-level property, exhibited across the whole experiment (reliable, 80% correct; unreliable, 50% correct), and is manipulated in Experiment 1. Difficulty is a stimulus-level property, defined by the color-proportion difference between the two stimuli, and determines how much the partner's gaze is needed. Goal congruency is a trial-level property, set anew by the auditory action context, and is manipulated in Experiment 2. We predict that heuristic weighting is strongest when the partner is reliable, the task is difficult, and gaze is goal-aligned, and is attenuated when gaze conflicts with the action plan. By targeting the two points separately, the two experiments test whether a single gaze-weighting heuristic operates across both, providing an ecological test of how reliability, difficulty, and goal congruency top-down regulate the use of gaze during joint action. Note that we distinguish two terms throughout. We use *gaze-weighting heuristic* for the cross-experiment construct, the flexible policy by which observers weigh a partner's gaze according to its expected utility for the joint task. We reserve gaze orienting for the narrow attentional phenomenon, the covert shift of attention to a gazed-at location indexed by target reaction times, which we measure in Experiment 2.

The two experiments differ in what the robot's gaze is validated against. In Experiment 1, we manipulate gaze-informative validity. The stimulus itself determines the correct response, so the robot's gaze is valid when it points toward the response the stimulus already specifies and invalid when it points toward the other action goal (bin in which objects are placed). Validity here is informative because it concerns a correct answer that exists independently of, and prior to, the gaze. In Experiment 2, we manipulate gaze spatial validity in the traditional sense. The target location is unpredictable and revealed only when the target appears, so the robot's gaze is valid when it coincides with where the target subsequently appears, and invalid otherwise. Validity here is spatial because it concerns a location that gaze cannot know in advance. When discussing both experiments together, we use the term “validity”, specifying informative or spatial on first use.

## 2 Experiment 1

Experiment 1 investigated whether task difficulty and the gaze reliability of the robot co-actor's gaze in a goal-oriented task influence deployment of the gaze-weighting heuristic. Building on proposals that cue reliability modulates attentional orienting to gaze cues (Wiese et al., 2012) and evidence that joint action framing modulates sensitivity to robot signals (Abubshait et al., 2020; Abubshait & Wiese, 2017), it is crucial to manipulate the consistency of gaze cues and the robot's performance, alongside task difficulty, to determine how gaze use is calibrated during real interactive exchanges. This experiment modified the paradigm from Abubshait et al. (2024). Here, participants performed an object-sorting task with the iCub humanoid robot. After viewing a colored stimulus on screen, they placed a cube on the left or the right depending on the dominant color of the stimuli presented on the screen. Notably, before participants responded, the robot was programmed to suggest a possible location using gaze and head orientation. With regard to cue reliability, we tested two groups: Group A (random) observed a robot that provided non-informative suggestions (50% random gaze reliability), while Group B (reliable) observed a robot that correctly indicated the target location on 80% of the trials (80% gaze reliability). We also manipulated the task difficulty, with easy and difficult trials. We hypothesized that the gaze-weighting heuristic would be calibrated by both reliability and difficulty, meaning that participants should rely on the robot's gaze more when it had proven reliable and when the task was challenging. Specifically, we expected that high gaze reliability would enhance reliance on the robot's gaze, resulting in an interaction between gaze reliability and gaze congruency in reaction times. Additionally, we expected greater reliance on the robot's gaze during difficult trials, when participants' own perceptual certainty was reduced and the heuristic value of a reliable partner's signal would be highest.

### 2.1 Methods & materials

#### 2.1.1 Participants

We conducted an a priori power analysis for the effect of *gaze informative validity*, using: (i) a moderate effect size (dz = 0.6), (ii) an α-error equal to 0.05, and (iii) a power level of 0.95 (as recommended by Cohen, 1992). This analysis yielded an adequate sample size of 24 per group. 49 participants were recruited for the experiment (36 female, 13 male, 41 right-handed, 7 left-handed, 1 ambidextrous, M = 23.12, SD = 4.38), divided into two groups. Group A (*N* = 25) interacted with a robot whose gaze shifts were randomized (i.e., non-informative regarding the correct response, 50% validity); Group B (*N* = 24) interacted with a robot whose gaze shifts were mostly informative (i.e., reliable regarding the correct response; 80% validity). Participants were unaware of the experiment's aims but were debriefed upon completion. All participants were paid a monetary compensation of 20 Euros. The experiment was conducted at the Italian Institute of Technology (IIT), where all experimental procedures involving the collection of behavioral data with healthy adult participants (procedures that do not involve any invasive or potentially dangerous methods) are approved by the Ethics Committee of the Liguria Region following the Code of Ethics of the World Medical Association (Declaration of Helsinki). Data were stored and analyzed pseudo-anonymously.

#### 2.1.2 Experimental design

Participants performed a classification task, during which they sat across from iCub with a 42-inch screen between them, placed horizontally; see Fig. 1. Participants were asked to sort a real physical object (i.e., blue or yellow cube) depending on its color into one of two real physical containers that were placed on the top corners of the screen. Each trial began with participants pressing a response button in front of them. As soon as the participant pressed the response button, two bars appeared laterally on the screen (i.e., one on the left side and one on the right side). Each of the two bars had a ratio of blue to yellow colors such that one color was more dominant than the other (e.g., the bar on the right was 70% blue and 30% yellow, whereas the bar on the left was 10% blue and 90% yellow). During the task and before the participants were allowed to place the cube on the correct side, iCub suggested where to place the cube with a combined head-and-gaze shift; this shift was either informative (valid) or non-informative (invalid) with the correct response. Participants got two points for a correct response or lost two points for an incorrect response.

**Fig. 1:**
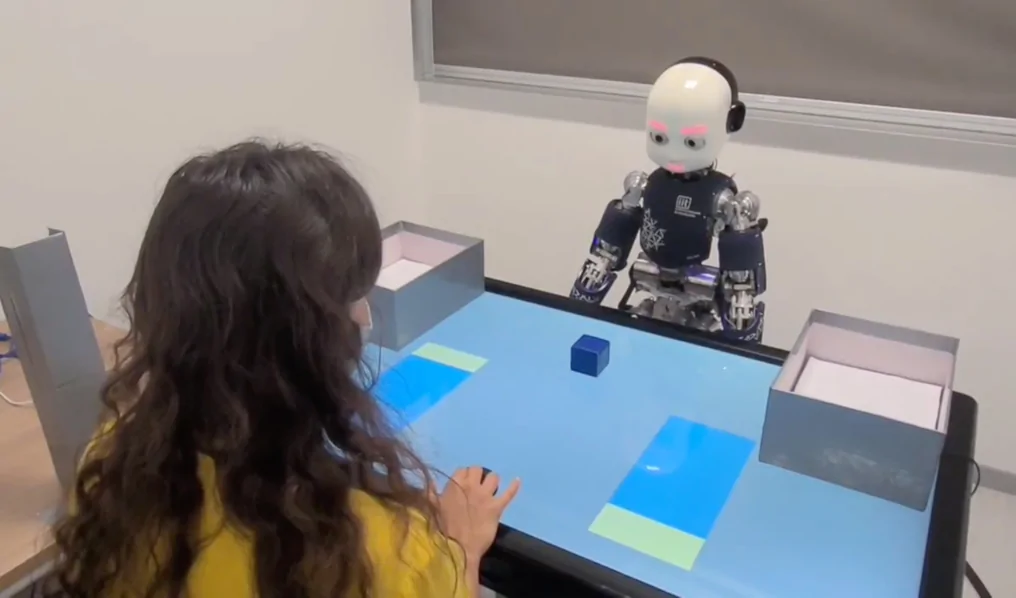
Experiment 1 setup. Participants sat down in front of the robot to perform the task.

We manipulated task difficulty by varying how similar the proportion of the two colors were for each bar. For instance, the right bar would be 70% blue, while the left bar would be 73% blue with a difference of only three points in this example. The smaller the differences between the two bars, the more difficult the object classification based on color should be: a difference in color predominance of 1% and 4% between bars was defined as “high-difficulty”; a difference in color predominance of 5% and 8% between bars was defined as “low-difficulty”.

#### 2.1.3 Task & procedure

After providing consent and filling in demographics questionnaires, participants sat across from iCub and were provided instructions for the task. At the beginning of each trial, participants took a cube from the dispenser, placed it over a yellow cross in the middle of the screen and then pressed and held a response button that was placed in front of them. Next, two bars appeared on the screen and remained there until response, one on the left and one on the right side of the cube. Each of the two bars had a blue-to-yellow ratio such that one color was more dominant than the other.

Participants then had to choose where to place the cube depending on the predominant color of the bars. For example, if the participants had a blue cube and the bar on their right was 70% blue and 30% yellow, and the bar on their left was 10% blue and 90% yellow, the correct position for the cube was the right container. After the bars appeared on the screen, iCub looked down for 2500 ms, then looked straight ahead at the participant (1800 ms) and then made a head/gaze shift (600 ms). Participants were instructed to keep the start button held until the robot made the gaze shift. This ensured that the robot's gaze was always displayed before participants could respond. Only then, they could take the cube from the table, and drop it inside the container. After placing the cube in the container, participants returned their hands to the starting position and pressed the black button again. Reaction times were measured from the moment participants stopped pressing the start button until the cube was dropped inside the container. After that, “correct” or “incorrect” feedback appeared on the screen for 1000 ms, corresponding to their task performance; see Fig. 2. Based on the correctness, +2 or −2 was added to the cumulative score, shown at the block's end. The experiment had 15 blocks, 8 trials per block, which adds up to 120 trials overall. After the task, participants were asked informally about the experiment and strategy and were debriefed. The experiment took around 45 min.

**Fig. 2:**
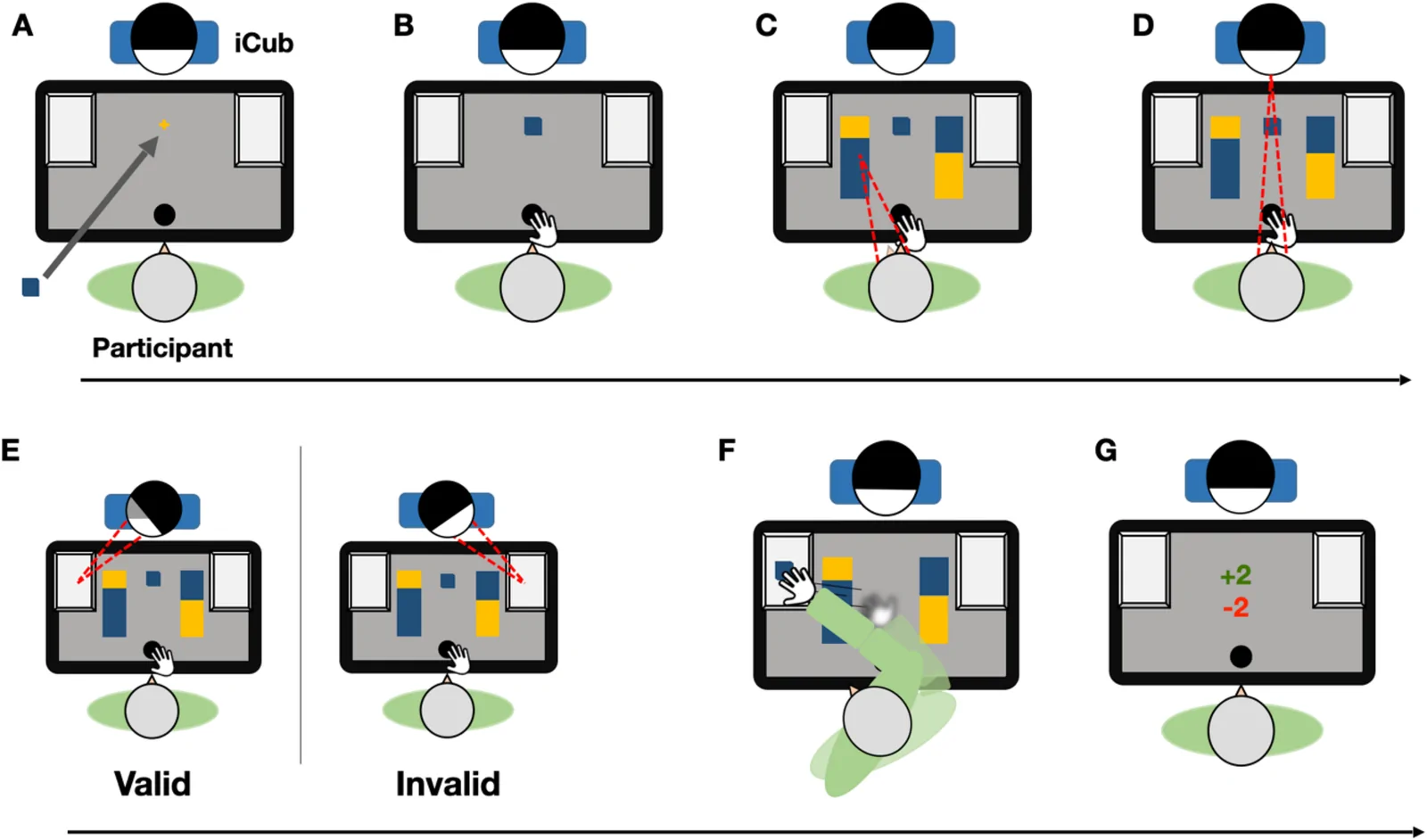
Schematic task sequence Experiment 1. The participant picked up a cube (A), placed it at the start position, and held the start button to begin (B). Two colored bars then appeared on the screen beside the containers, indicating the blue–yellow proportions. Participants evaluated the bars and prepared their choice while continuing to hold the button (C). After looking straight ahead (D), the robot shifted head and gaze toward one bin, providing a suggestion that could be correct or incorrect (E). The participant dropped the cube into a container depending on the side with the higher proportion matching the cube color (F) and then received feedback (G). (For interpretation of the references to color in this figure legend, the reader is referred to the web version of this article.)

#### 2.1.4 Apparatus

Participants sat approximately 120 cm in front of the robot. Details regarding robot control can be found in the Supplementary material. Between them, a 42″ multi-touch screen (1099.4 × 634.0 × 36.8 mm, 60 Hz) screen was horizontally placed 90 cm from the floor; see Fig. 1. This arrangement allowed us to use the table for stimulus presentation and shared space/table. At the left and right extremes of the screen, two boxes were placed close enough for participants to drop the cube. Participants' responses were collected using three physical Logitech Gaming Kit buttons, one under each box to detect the drop of the cube and one in the middle close to the participant. These latter buttons started the trial and allowed us to measure the starting of the reaching movement. PsychoPy (Peirce et al., 2019) and the middleware YARP were used to control and synchronize the robot's movements (details in the Supplementary material).

#### 2.1.5 Analysis

We analyzed the error rates and reaction times in a 2 × 2 × 2 mixed ANOVA with the within-participants factors *Difficulty* (high vs. low) and *Gaze Informative Validity* (valid vs. invalid) and the between-participants factor *Reliability* (random vs. reliable). Planned simple contrasts (Bonferroni corrected) were used to compare conditions. Error rates were calculated as the proportion of incorrect responses for each participant in each condition. Reaction times were preprocessed by first removing incorrect trials, then excluding trials with reaction times exceeding 2 s, and finally removing trials with reaction times more than 4 SD beyond each participant's mean within each condition. The 2-second ceiling was applied first to remove implausibly slow responses prior to standardization, making the subsequent SD-based filter a within-participant trimming procedure on the remaining distribution. Error rate was the primary dependent variable, as reaction time in our paradigm reflects movement execution rather than decision time. Reaction-time analyses are reported in the Supplementary Materials.

### 2.2 Results

#### 2.2.1 Error rates

A 2 (*Reliability*: random vs. reliable) × 2 (*Validity*: valid vs. invalid) × 2 (*Difficulty*: low vs. high) mixed ANOVA was conducted on error rates (see Table 1).

**Table 1:** Error rates (percentage) and standard deviations per condition, Experiment 1.

Reliability	Informative validity	Difficulty	Mean	SD
Random	Valid	High	0.20	0.157
	Invalid		0.22	0.161
	Valid	Low	0.04	0.082
	Invalid		0.05	0.106
Reliable	Valid	High	0.16	0.125
	Invalid		0.31	0.239
	Valid	Low	0.03	0.082
	Invalid		0.04	0.136

Analyses yielded no main effect of *Reliability*, *F*(1, 47) = 0.45, *p* = .505, partial η^2^ = 0.01, but significant main effects of *Validity*, *F*(1, 47) = 20.76, *p* < .001, partial η^2^ = 0.31, and *Difficulty*, *F*(1, 47) = 325.21, *p* < .001, partial η^2^ = 0.87. The *Reliability × Validity* interaction was significant, *F*(1, 47) = 8.90, *p* = .005, partial η^2^ = 0.16, whereas the *Reliability* × *Difficulty* interaction was not, *F*(1, 47) = 1.88, *p* = .177, partial η^2^ = 0.04. There was a significant V*alidity* × *Difficulty* interaction, *F*(1, 47) = 14.41, *p* < .001, partial η^2^ = 0.23, which was qualified by a significant three-way interaction among *Reliability*, V*alidity*, and *Difficulty*, *F*(1, 47) = 11.16, *p* = .002, partial η^2^ = 0.19. These results indicate that error rates were higher under high difficulty and on invalid trials, and that the size of the validity effect varied with difficulty and differed between groups; see Fig. 3.

**Fig. 3:**
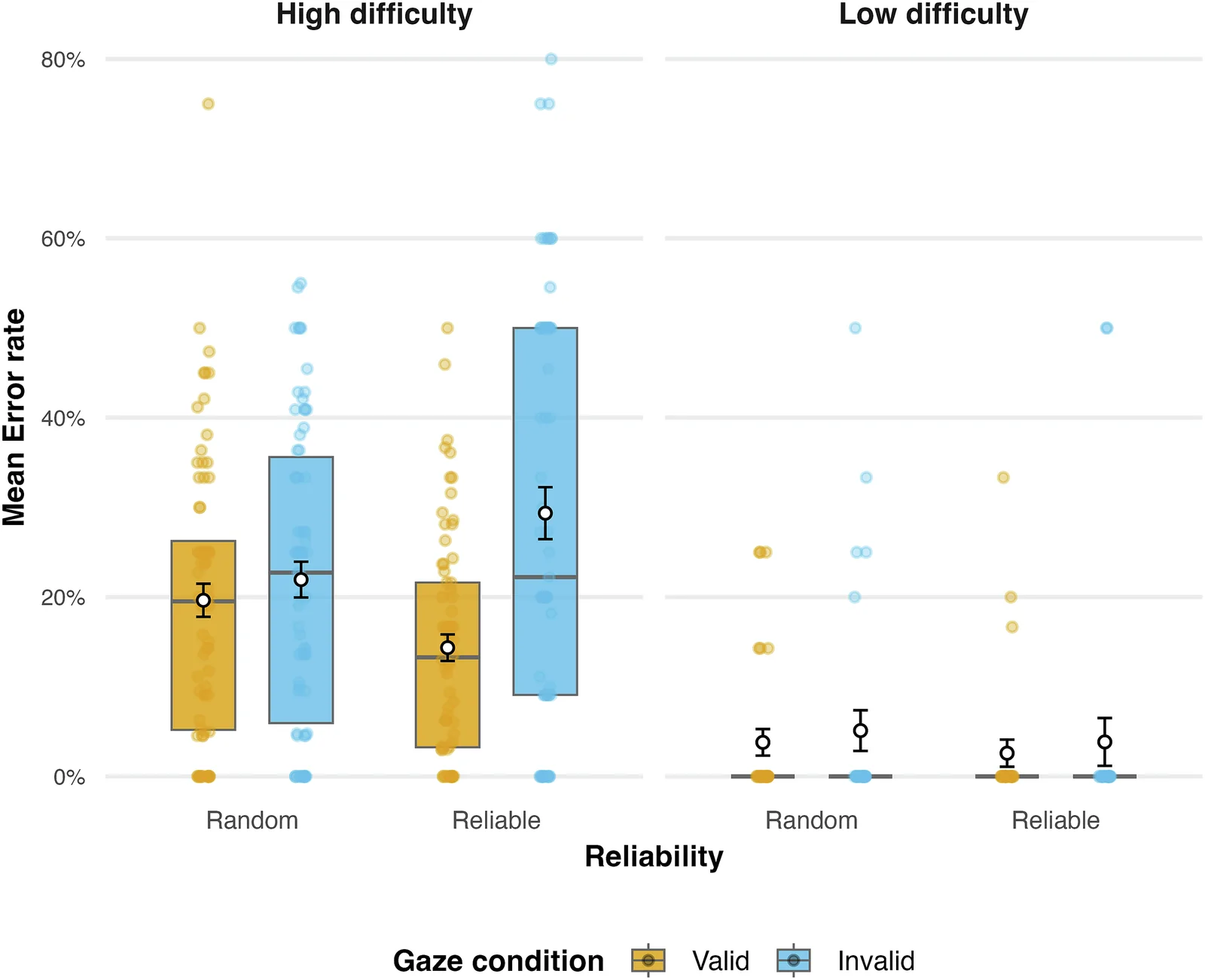
Error rates by reliability, validity, and difficulty. Error rates were higher on high than low difficulty trials and on invalid compared to valid trials. The validity effect depended on task difficulty and differed between reliability groups: only the reliable group showed a validity effect under high difficulty. Groups differed only in the high-difficulty invalid condition, in which the reliable group made more errors than in all other conditions. In each panel, shaded boxes span the interquartile range (25th to 75th percentiles) of per-participant error rates, with the internal horizontal line indicating the median. White points show the group mean and black error bars show ±1 standard error of the mean. Semi-transparent points show individual participant error rates.

Simple effects post hoc tests (Bonferroni-adjusted; Kenward–Roger df) of validity within each Reliability × Difficulty showed a reliable validity effect only in the reliable group under high difficulty: fewer errors on valid than invalid trials, Δ = −0.147, 95% CI [−0.198, −0.095], *t*(351) = −5.60, *p* < .001. The same contrast was not significant for the random group at high difficulty, Δ = −0.022, 95% CI [−0.073, 0.028], *t*(350) = −0.87, *p* = .388, nor for either group under low difficulty (|*t*| ≤ 0.39, *ps* ≥ .698).

Between-reliability group comparisons within each Validity × Difficulty indicated that groups differed only in the high-difficulty invalid condition, with the random group committing fewer errors than the reliable group, Δ = −0.087, 95% CI [−0.141, −0.033], *t*(211) = −3.17, *p* = .002. No group differences emerged for high-difficulty valid condition or for either validity under the low-difficulty condition (|t| ≤ 1.38, ps ≥ .158).

Difficulty effects within each Reliability × Validity were robust across conditions, as error rates were higher on high- versus low-difficulty for the random group on the valid condition, Δ = 0.160, 95% CI [0.094, 0.225], *t*(358) = 4.79, *p* < .001; the reliable group on the valid condition, Δ = 0.127, 95% CI [0.060, 0.195], *t*(355) = 3.71, *p* < .001; the random group on the invalid condition, Δ = 0.167, 95% CI [0.102, 0.231], *t*(358) = 5.06, *p* < .001; and the reliable group in the invalid condition, Δ = 0.266, 95% CI [0.196, 0.336], *t*(354) = 7.46, *p* < .001.

Finally, difference-of-differences tests confirmed that the Reliability × Validity interaction was present at the high-difficulty condition. The validity effect was larger in the reliable than the random group, Δ = 0.124, 95% CI [0.052, 0.197], *t*(351) = 3.39, *p* < .001, but absent at low-difficulty, Δ = −0.007, 95% CI [−0.120, 0.106], *t*(359) = −0.13, *p* = .900. Together, these findings indicate that difficulty had a significant impact on performance across conditions, while validity effects were only reliable in reliable group under high difficulty conditions. Differences between groups were isolated to the most challenging condition: invalid, high-difficulty trials. Note that reaction times, which in our paradigm reflect movement execution rather than decision time, are reported in full in the Supplementary Materials (Fig. S1); the pattern was broadly consistent with the error-rate results.

### 2.3 Discussion Experiment 1

Experiment 1 investigated whether people deploy the gaze-weighting heuristic as a function of a robot co-actor's reliability and task difficulty during a goal-directed sorting task. Participants placed colored cubes left or right while iCub provided a head/gaze suggestion (i.e., a cue) that was either valid (informative regarding the correct response) or invalid (uninformative). The reliability of those suggestions was manipulated between groups (Random: 50% reliability vs. Reliable: 80% reliability) and within participants, we manipulated difficulty (low vs. high). We predicted stronger validity advantage (better performance on valid than invalid trials) when the robot was reliable, especially under high-difficulty. The accuracy results showed that participants strongly relied on the robot's signals in their decisions. Errors increased with difficulty, and a validity advantage appeared only for the reliable group under high difficulty, with group differences limited to the high-difficulty invalid condition. In other words, participants relied on the robot's gaze when the cue had proven accurate and when perceptual demands were high. Critically, participants in the reliable group made more errors on high-difficulty invalid trials than the random group, which is a signature of genuine reliance rather than random responding. The *cost of reliance* indicates that participants weighted the robot's gaze most when their own perceptual certainty was low and the robot had proven trustworthy. Only participants who were weighting the gaze highly (and thus taking it into account) could be misled by it during invalid trials. By contrast, when suggestions were uninformative (random) or the discrimination was easy, weighting the robot's gaze offered no benefit, consistent with a utility-weighting, context-sensitive process. Reliance on the robot's gaze was top-down modulated by the interaction between reliability and task demands, rather than applied uniformly across conditions.

This flexibility is the central point. The robot's gaze causally influenced participants' decisions, valid gaze facilitated performance and invalid gaze impaired it. This influence emerged under high difficulty, was higher with the robot's demonstrated reliability, and produced its clearest cost when a reliable partner's gaze turned out to be invalid. Top-down factors thus did not merely add to a stimulus-driven response; they determined whether the robot's gaze was weighted at all. The gaze functioned as an actively weighted signal. Because our task contained no attentional measure, we remain agnostic as to whether this influence operated through attentional orienting toward the gazed-at location, through a decision-level weighting of the gaze as evidence, or through both. Our claims for Experiment 1 are therefore confined to the flexible, top-down influence of gaze on decision-making, and we return to the attentional level in Experiment 2, where target reaction times provide the appropriate measure. These findings also extend previous work (Abubshait et al., 2024) by demonstrating that the reliability-by-validity effect scales to a more complex, continuous interaction where participants must integrate the robot's signals with their own uncertain perceptual evidence in real time.

Additionally, our results provide strong evidence for a joint action perspective on gaze use. During collaborative tasks, partners continuously form and update models of each other's reliability (Sebanz & Knoblich, 2009; Vesper et al., 2017). These findings show that this partner modeling directly shapes the deployment of the gaze-weighting heuristic: participants who experienced a reliable co-actor relied on its signals into their decisions, whereas those with an unreliable partner did not. This calibration occurred despite identical perceptual demands and identical robot behavior at the trial level. The only difference was the statistical structure of reliability across the experiment. The differences between the two groups indicate that reliance on the robot's gaze was sensitive to its reliability, consistent with predictive models of social attention (Kuhn et al., 2018; Stephenson et al., 2021).

Our findings also extend screen-based work showing rapid discounting of unreliable observed gaze and greater recruitment of social cues under load (e.g., Dalmaso et al., 2020; Wiese et al., 2012) to a live, second-person exchange in which the robot's gaze was temporally coupled to participants' actions and had immediate consequences for task success. Remarkably, the interactive nature of the task meant that participants were not passive observers but active co-actors whose performance depended on successfully integrating their own perceptual judgments with the robot's suggestions, a hallmark of joint action coordination (Knoblich et al., 2011).

Taken together, the findings from Experiment 1 show that in an interactive, goal-directed task, the gaze-weighting heuristic is selectively deployed based on partner reliability and task demands: people count on reliable gaze under high-demand tasks or down-weight it otherwise. This selectivity supports a predictive, utility-weighting view of gaze use during joint action and offers a concrete design principle for transparent, gaze-aware collaborative robots, thereby making the validity evident and the costs of misuse salient. From a joint action perspective, these findings suggest that successful human-robot collaboration requires robots to establish and maintain reliability, as humans calibrate their reliance on robotic partners' signals. However, reliability observed across the interaction is only one dimension along which the heuristic might be calibrated. A co-actor's gaze also carries moment-to-moment meaning tied to what the partner's upcoming actions are. It is unclear whether the heuristic is sensitive to this trial-level, goal-related dimension independently of reliability in a single task. Experiment 2 addresses this by embedding gaze within a richer action sequence in which goal-related expectations are established on each trial, testing the gaze-weighting heuristic in a different setting.

## 3 Experiment 2

During joint action, partners form expectations about action sequences (Sebanz & Knoblich, 2009), and gaze that aligns with these expectations should be more readily used to guide attention, whereas gaze that conflicts with action goals may be attenuated. Experiment 2 is an adaptation of Perez-Osorio, Müller, Wiese and Wykowska, 2015, Perez-Osorio, Müller and Wykowska, 2017, embedding a gaze-cueing task within an object handover sequence to examine whether participants' use of the robot's gaze depends on whether the gaze aligns with the expected action goals. This experiment tested whether gaze orienting is gated by goal congruency, a situational factor arising from action expectations established on a trial-by-trial basis. Participants stood in front of the iCub robot with two bottles in front of them. On each trial, a contextual auditory cue (something to drink or to do the laundry) established an action goal, making one bottle more relevant than the other. Then the robot looked either at the object that matched the action goal (goal-congruent gaze) or at the alternative object (goal-incongruent gaze). Goal congruency is thus a trial-level property of where the robot's gaze is directed relative to the current action context, and not an inference about whether the robot and participant share a common intention. Subsequently, a target letter briefly appeared beneath one of the bottles, and participants reported the letter while ignoring a distractor symbol, following the standard gaze-cueing procedure. Note that the robot's gaze direction could be either spatially valid (target appeared at gazed-at location) or spatially invalid (target appeared at the opposite location), crossed with whether the gazed-at object was goal-congruent or goal-incongruent with the action context. To control where participants looked during the task and to probe the underlying attentional mechanisms, we recorded eye movements. Eye fixation analyses assessed whether expectations about action sequences influenced gaze shifts toward the robot's face and subsequent orienting to the cued objects.

We aimed to test whether previous findings can be replicated in real joint action settings, and specifically whether gaze orienting is modulated by action congruency when gaze is embedded in meaningful, goal-directed interactions with an embodied humanoid robot. Whereas Experiment 1 manipulated reliability (a partner characteristic), Experiment 2 manipulated goal alignment (a situational characteristic), testing whether both dimensions jointly shape heuristic deployment. In line with the heuristic framework and previous findings, we hypothesized that the gaze-weighting heuristic would be more strongly deployed when the robot gaze aligned with the established action goal. We expected faster and more accurate responses to targets appearing at goal-congruent locations following the robot's gaze, with attenuation of the standard spatial validity effect when gaze conflicted with action expectations.

### 3.1 Methods & materials

#### 3.1.1 Participants

We conducted an a priori power analysis to determine the proper sample size for the Experiment, specifically for the effect of congruency on gaze-cueing. The variables we used were (i) a medium effect size (*d*_*z*_ = 0.50), (ii) an α-error equal to 0.05, and (iii) a power level of 0.80 (as recommended by Cohen, 1992). This analysis yielded an adequate sample size of 33. 41 participants were recruited for the experiment to obtain 39 usable data sets (two participants were excluded due to error rates exceeding 30% in at least one experimental condition). The additional participants were recruited to compensate for any potential low-quality data in the eye-tracking recording and potential effects of real-life interaction. Eye-tracking data quality was assessed based on achieving at least 80% of successfully tracked samples during the gaze-cueing part of the task. All 39 retained participants (Mage = 26.06, SD = 5.76, 22 women, 17 men, 34 right-handed, 5 left-handed) had equal or greater valid eye-tracking data samples, had normal or corrected-to-normal vision, including normal color vision, and none reported a history of neurological disease. They were unaware of the experiment's aims and had not previously participated in a similar experiment.

Participants provided their informed consent and were compensated for their participation. The experiment was conducted at the Italian Institute of Technology (IIT), where all experimental procedures involving the collection of behavioral data with healthy adult participants (procedures that do not involve any invasive or potentially dangerous methods) are approved by the Ethics Committee of the Liguria Region following the Code of Ethics of the World Medical Association (Declaration of Helsinki). Data were stored and analyzed pseudo-anonymously.

#### 3.1.2 Experimental design

We used a 3 × 2 design with the factors *Action Congruency* (congruent vs. incongruent vs. neutral) and *Spatial Validity* (valid vs. invalid) as within-participants factors. On each trial, an auditory cue established the action context (“something to drink”, “something to do the laundry”, and “the weather is nice today”), which specified an action goal and thereby the expected action, taking the object that satisfies the goal, and the bottle that action requires (the beverage bottle, the fabric softener or none in the neutral context). Action Congruency was defined as whether iCub's gaze was directed toward the bottle required by the expected action (e.g., the juice bottle was congruent in the context “something to drink”), toward the other bottle (incongruent), or, when the context specified no goal (for example, “the weather is nice”), toward either bottle (neutral). Spatial validity was defined solely with respect to iCub's gaze direction. The target appeared at the gazed-at location on valid trials and at the opposite location on invalid trials, with equal probability across all congruency conditions.

Furthermore, the two factors are fully crossed, as the target appears at the gazed-at location in 50% of trials in each congruency condition. Because of that, iCub's gaze was not predictive of target location, and neither the side of presentation nor the identity of the bottle predicted where the target would appear. The target appeared only after the gaze shift had been completed. To enhance ecological validity and increase interaction engagement, iCub grasped one of the bottles and handed it to the participant.

Participants were asked to fixate on the robot's face until a response was given, after which they were encouraged to move their eyes freely. We measured fixations to better understand the attentional mechanisms engaged in the task. Importantly, eye tracking also allows us to evaluate whether participants looked at iCub's face and whether predictions regarding action sequences impact gaze following and bottle handling.

#### 3.1.3 Task & procedure

Participants were informed that they would assist iCub in practicing speech recognition and grasping during the experiment. To do so, they were asked to evaluate whether iCub took the correct bottle depending on the request randomly generated by the computer (presented via speakers). This provided a framing for the handover. A typical trial began with two transparent bottles placed on a translucent table, atop a dark grey placeholder. Then, sentences setting the action context (“something to drink”, “something to do the laundry,” or “the weather is nice” in Italian) were broadcasted via speakers while iCub looked at the participant for 2000 ms. Afterwards, via projection from below, one of the bottles turned into the beverage bottle (yellow) and the other one into the fabric softener bottle (blue). Subsequently, iCub looked either to the left or the right bottle. This temporal order was necessary for the congruency manipulation. Because the bottles were transparent and visually identical until the projection assigned their identities, a gaze shift occurring before the reveal would have been directed toward an unidentified container and could have been neither congruent nor incongruent with the action goal. The auditory context established the goal, and the projection established which bottle satisfied it, so that iCub's subsequent gaze shift could be interpreted against a fully specified action context, as it would be in natural joint action.

Subsequently, iCub looked either to the left or the right bottle. iCub's gaze shift (eyes and neck moving simultaneously) took 1200 ms to complete (800 ms for eye movement, 1200 ms for neck movement). Only after the movement was completed a target letter (either V or T) appeared for 300 ms under one of the bottles. Meanwhile, on the opposite side, an octothorp (“#”) was presented. This was done to avoid bottom-up effects due to the abrupt onset of the target on one of the sides. Participants were asked to respond to the target identity by pressing the letters “z” for “V” and “x” for “T” with the left index and middle finger using a keyboard (QWERTY layout). Participants were allowed to respond from target onset. After target presentation, the target was masked with an octothorp until a response was made or the timeout criterion of 1300 ms was reached, whichever came first. Participants had to respond to the letter on the screen as accurately and quickly as possible. After the target offset, the robot looked at the participant for 1000 ms, then gazed at one of the bottles, approached it, grasped it, and offered it to the participant. The bottle iCub selected for the handover was determined independently of the pre-target gaze shift. Across the experiment, iCub handed over the goal-appropriate bottle on half of the trials, and this was not contingent on whether its earlier gaze had been congruent or incongruent with the action context. The handover therefore served as a balanced cover task that carried no systematic information about the reliability of iCub's gaze, so that action congruency remained a trial-level property of the gaze rather than a cumulative signal of partner reliability. Participants were asked to take it and put it back on the placeholder (colored circle); see Fig. 4.

**Fig. 4:**
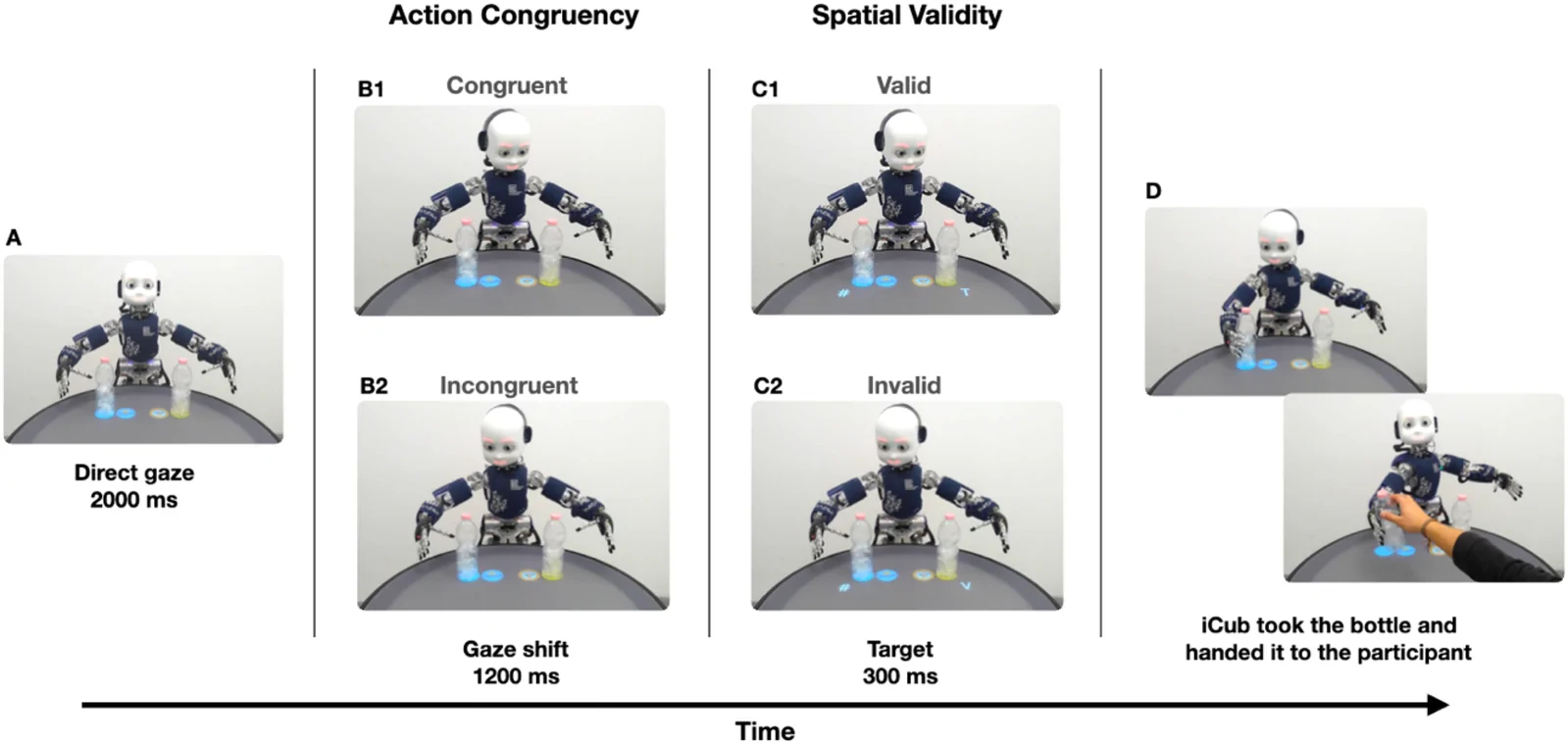
Schematic task sequence Experiment 2. Example of “something to drink” context. Yellow represents orange juice (action congruent) and blue represents fabric softener (action incongruent). The context was presented auditorily, and the robot's gaze was directed to the participant (A), then the robot made a gaze shift congruent (B1) or incongruent (B2) with the action context. The target appeared in the spatially valid (C1) or invalid (C2) location. Participants were asked to fixate on the robot's face until this point. Then iCub picked up one of the bottles and gave it to the participant (D). Before the end of the trial, participants were asked whether the robot selected the correct bottle based on the action context. (For interpretation of the references to color in this figure legend, the reader is referred to the web version of this article.)

At the end of each trial, participants evaluated whether iCub took the correct bottle depending on the request made at the beginning. The question was “Did iCub take the correct bottle?” appeared at the center of the table. Here, “correct” refers to whether the bottle iCub selected matched the auditory action context presented at the start of the trial. The question was unrelated to the target letter or its location. It served as an attention and comprehension check, ensuring that participants processed the action context on each trial and attended to iCub's action, and it provided the framing for the handover task. Participants responded to the question by using their right hand over the number pad on the right side of the keyboard. The options were “1” for “yes”, “2” for “no,” and “3” for “NA”. NA were the letters for “does not apply” in Italian, used when the sentence was related to the weather. The questions were presented on the screen for up to 5000 ms or until a response was given. All conditions were pseudo-randomized across trials; additionally, the side on which each bottle was presented (left or right), the target type (V or T), and the target position (low or high) were also pseudo-randomized. The total number of trials was 96, presented in 8 blocks of 12 trials each, the six conditions (3 congruency × 2 validity) were presented 16 times. All congruency conditions were distributed equally. All participants received identical instructions and had a block of practice with the robot and the eye-tracking glasses to facilitate adaptation to the task. Each experimental session lasted 90 min, which included practice, calibration, and the actual experimental trials.

Participants responded to the target with their left hand, which remained in a fixed position on the keyboard throughout the trial for three reasons: (a) the right hand free to receive the bottle during the handover and to answer the context question on the number pad, (b) it restricted hand movements during the cue-to-target interval, and (c) it removed any need to look down at the keyboard during the task, which would have drawn fixation away from iCub's face during the critical gaze/head shift period.

#### 3.1.4 Apparatus

Participants interacted with the humanoid robot iCub (Metta et al., 2010). The experiment was performed in an isolated and noise-attenuating room. Participants stood in front of iCub at approximately 85 cm. The iCub was placed behind a translucent table (ø 83 cm, 91 cm height) illuminated from below by a projector (60 Hz refresh rate). iCub's eyes were located about 130 cm from the ground and about 36 cm above the table. Stimuli were projected on a translucent area of about 0.32 m^2^. Four circles, each with a diameter of 7 cm were projected at 61 cm from the border of the table, equidistant from the center of the midline. On each side, there were two circles: one more proximal to the midline, used to display the liquid type per bottle (drink vs. fabric softener) and another one more distal and placed below a transparent bottle (measuring 24 cm, with a diameter of 6.5 cm) that displayed a solid color matching the liquid type (drink in yellow and fabric softener in blue). The target stimuli were letters “V” or “T” located below the bottles at 17.7 cm from the midline; each letter was 4 × 3 cm in bold. The action context (drink, laundry or neutral) was set via the auditory presentation of different sentences at the beginning of each trial. The sentences were created using the free online Text Speech (TTS) service with natural-sounding voices (http://www.fromtexttospeech.com/). All the sentences were matched for volume (45 dB).

Participants' eye movements were measured using the Tobii Pro Glasses 2, a lightweight, commercial mobile eye tracker able to record ocular data at a frequency of up to 100 Hz. The plugin implemented to control the Tobii Pro Glasses 2 enabled the start and stop of recordings for each participant and calibrated the eye tracker at the beginning of each block using a single calibration routine. Calibration accuracy was verified using a pass/fail criterion in the glasses recording software, and recalibration was performed if calibration failed. We monitored participants' gaze behavior during the gaze-cueing task from the initiation of gaze-head movement to the response to the letter to determine whether participants followed iCub's gaze. We processed the data offline using the Tobii Pro Lab software for the eye-tracking analysis.

OpenSesame and the iCub middleware YARP (Metta et al., 2006) were used to control the robot's movements (details in the Supplementary material). OpenSesame (Mathôt et al., 2012) was used to control and synchronize iCub's movements, and to collect eye tracking and performance data (with the additional help of custom-made plugins).

### 3.2 Analysis

#### 3.2.1 Behavioral data

Error rates and median reaction times were calculated for the letter discrimination task, similar to Perez-Osorio, Müller, Wiese and Wykowska, 2015, Perez-Osorio, Müller and Wykowska, 2017. Those measures were entered into a 3 × 2 ANOVA with *Action Congruency* (congruent vs. incongruent vs. neutral) and *Spatial Validity* (valid vs. invalid) as within-participants factors. Values reported in the text represent the mean of these individual medians across participants. Median reaction times were calculated for each experimental condition and per participant and only correct trials were included. Medians provide a more robust estimate of central tendency than the mean and were used in the original publication. Observers' error rates in the context question (choosing the right action context) were less than 2% on average (M = 1.14%, SD = 2.17). We did not have enough data to run statistical analyses on these responses. This high accuracy reveals that participants attended to the task. Mauchly's test indicated that the assumption of sphericity was met for all effects involving factors with more than two levels, so uncorrected values are reported.

#### 3.2.2 Eye tracking data

To verify that participants maintained fixation on the robot's face during the task and to rule out overt eye movements as a confound in interpreting reaction-time effects, we conducted a fixation-based analysis of gaze data exported from Tobii Pro Lab. The fixations were mapped to Areas of Interest (AOIs) using automated mapping via Tobii Pro Lab's I-VT gaze filter. The filter uses a 100°/s velocity threshold and is intended for dynamic recordings, fixation defaults include a 60 ms minimum fixation duration, a 75 ms maximum time between fixations (for merging), and a 0.5° maximum spatial merge distance. Noise reduction is applied with a moving-median filter, interpolation is disabled, and eye selection averages both eyes.

Six predefined AOIs were defined: Robot Face (primary AOI of interest), Robot Body, Bottle Left, Bottle Right, Target Left, and Target Right. The AOIs were selected manually over a static picture from the perspective of the participant (See Fig. 6). The dataset was filtered to include only fixations, which had already been classified by Tobii Pro Lab using its default event detection algorithms. Per fixation, we identified whether it landed within a given AOI and calculated the following metrics per AOI: total number of fixations, total fixation duration, mean fixation duration, and their respective percentages relative to all fixations.

Because fixations are nested within trials and participants, we used models that account for the hierarchical structure of the data. A linear mixed-effects model with fixation duration as the dependent variable, AOI as a fixed effect and random intercepts for participants was used to test for different fixation durations across AOIs. Post-hoc comparisons were conducted using Tukey's HSD. To examine whether fixation counts differed between AOIs, a Poisson generalized linear mixed model (GLMM) with random intercepts for participants was fit with fixation count as the dependent variable and AOI as the categorical predictor. This model was selected because fixation count data are discrete and non-normally distributed. Estimated marginal means were used for pairwise comparisons between AOIs, with *p*-values adjusted for multiple testing.

### 3.3 Results

#### 3.3.1 Behavioral data

Participants responded to the letter discrimination task with a mean *error rate* of 5.40% (SD = 6.60). The repeated-measures 3 × 2 ANOVA on error rates revealed no significant main effect of Action Congruency, F(2, 76) = 3.10, *p* = .051, partial η^2^ = 0.075, and no significant main effect of Spatial Validity, F(1, 38) = 0.94, *p* = .339, partial η^2^ = 0.02. The Action Congruency × Spatial Validity interaction was significant, F(2, 76) = 4.22, *p* = .018, partial η^2^ = 0.10. Planned post-hoc comparisons showed a significant difference between congruent (4.16) and neutral (6.41), *t* (38) = −2.83, *p* = .022, 95% *CI* [−0.043, −0.003], Bonferroni corrected.

The repeated measures 3 × 2 ANOVA of the median reaction times revealed a main effect of *Validity* (*F*(1, 38) = 8.96, *p* = .005, partial η^2^ = 0.19), with participants responding faster on valid trials (*M* = 446 ms; *SD* = 140 ms) than on invalid trials (*M* = 462 ms; *SD* = 137 ms); the main effect of *Action Congruency* was not significant (*F*(2, 76) = 2.44, *p* = .093, partial η^2^ = 0.06), see Table 2.

**Table 2:** Reaction times (ms) and standard deviations by action congruency and validity, Experiment 2.

Action congruency	Valid		Invalid	
	Mean	SD	Mean	SD
Congruent	424	126	466	137
Incongruent	463	149	448	135
Neutral	450	145	473	138

Notably, there was a significant *Action Congruency* × *Validity* interaction effect (*F*(2, 76) = 10.74, *p* = .00007, partial η^2^ = 0.22), suggesting that the effect of spatial validity differed across action congruency conditions. Planned comparisons (Bonferroni corrected *t*-tests) revealed that congruent valid (*M* = 424 ms; *SD* = 125 ms) was significantly faster than incongruent valid (*M* = 463 ms; *SD* = 149 ms, *p* = .003), congruent invalid (*M* = 466 ms; *SD* = 137 ms, *p* = .001), and neutral invalid (*M* = 472 ms, *SD* = 137 ms, *p* = .001).

To directly quantify the magnitude and direction of gaze orienting within each action context and test whether it differed from zero, gaze-cueing effects were calculated by subtracting the median reaction times for valid trials from the median reaction times of the invalid trials (for each experimental condition). Positive values indicate that participants followed the robot's gaze direction, while negative values indicate that participants oriented their attention to the location opposite of where the robot was looking. Paired-sampled *t*-tests of the gaze-cueing effects showed significant differences between the congruent (*M* = 41 ms; *SD* = 59 ms) the and incongruent (*M* = −15 ms; *SD* = 50 ms) action contexts (*t* (38) = 4.57, *p* = .00001, 95% *CI* [25.691, 87.770]), as well as between the incongruent (*M* = −15 ms; *SD* = 50 ms) and neutral (*M* = 22 ms; *SD* = 59 ms) action contexts (*t* (38) = −3.401, *p* = .004, 95% *CI* [−65.99, −10.02]); see Fig. 5.

**Fig. 5:**
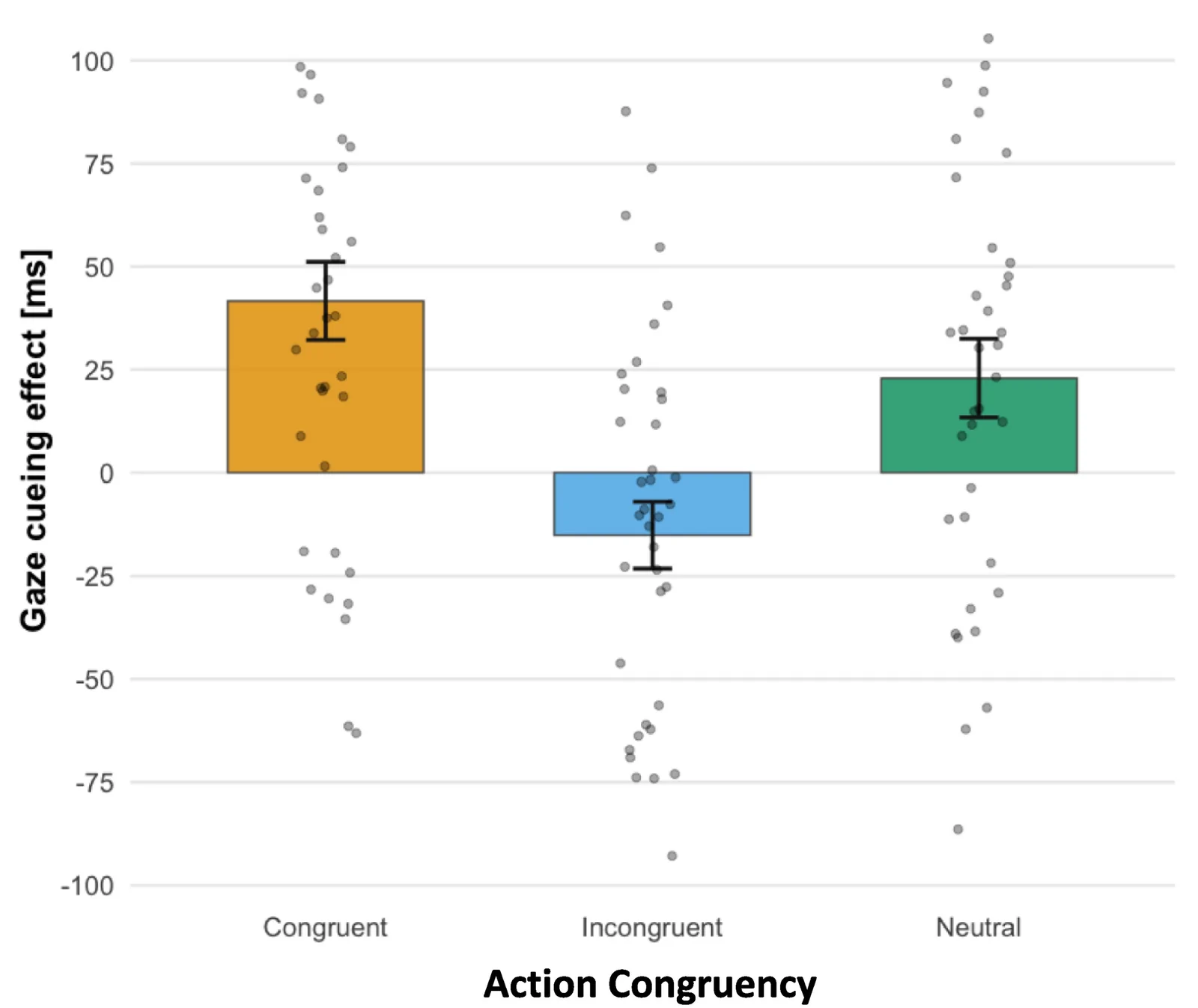
Gaze-cueing effects across action congruency conditions (context congruent, context incongruent, neutral context). Gaze cueing was positive (participants followed the robot's gaze) in the congruent and neutral contexts, but absent in the incongruent context. The effect was larger for congruent relative to neutral, with both exceeding the incongruent context.

#### 3.3.2 Eye tracking data

##### 3.3.2.1 Instruction compliance

To verify that participants maintained central fixation as instructed during the critical gaze-cueing period, we analyzed fixation patterns across all predefined AOIs. A linear mixed-effects model with fixation duration as the dependent variable, AOI as a fixed effect, and random intercepts for participants revealed a significant effect of AOI on fixation duration, *F*(5, 15285.85) = 284.28, *p* < .001, partial η^2^ = 0.09).

Consistent with task instructions, participants predominantly fixated the robot's face: 78.84% of all fixations and 92.17% of total fixation duration were directed to the robot face (M = 3373.62 ms per fixation), significantly exceeding all other AOIs (all ps < .001, Tukey HSD). Critically, bottles and targets each received fewer than 6% of fixations (Bottle Left: 5.34%, Bottle Right: 5.00%, Target Left: 0.43%, Target Right: 0.49%), confirming that participants maintained central fixation throughout the gaze-cueing sequence; see Table 3. Among the remaining AOIs, Target Right yielded longer fixations than Bottle Left and Right, and Robot Body (all ps < .001). Target Left did not differ from Bottle Left (*p* = .279) or Bottle Right (*p* = .246), but exceeded Robot Body (*p* < .001). Bottle Right fixations were longer than Bottle Left (*p* < .001).

**Table 3:** Fixations per AOI in milliseconds. Fixation metrics per AOI. All the metrics are highest for the robot face.

AOI	% Fixations	% Duration	Mean duration per fixation	SD_duration
Robot Face	78.84	92.17	3373.62	3443.92
Robot Body	9.91	3.40	988.87	1069.88
Bottle Left	5.34	2.13	1153.41	964.56
Bottle Right	5.00	1.90	1099.27	858.53
Target Right	0.49	0.23	1340.42	1027.43
Target Left	0.43	0.17	1126.81	765.94

A Poisson generalized linear mixed model examining fixation counts confirmed this pattern, χ^2^(5) = 31,171,000, *p* < .001. Relative to Bottle Left (reference category), fixation counts were significantly higher for Robot Face (β = 2.693, SE = 0.001, z = 2353.73, *p* < .001) and Robot Body (β = 0.619, SE = 0.001, z = 450.66, *p* < .001), whereas fixation counts were significantly lower for Bottle Right (β = −0.066, SE = 0.002, z = −41.31, *p* < .001), and substantially lower for Target Left (β = −2.526, SE = 0.004, z = −620.83, *p* < .001), and Target Right (β = −2.380, SE = 0.004, z = −625.64, *p* < .001). These findings confirm that participants followed task instructions to maintain fixation on the robot's face during the critical gaze-cueing window. This validates that the observed reaction time effects reflect covert attentional orienting rather than overt eye movements to the cued locations; see Fig. 6.

**Fig. 6:**
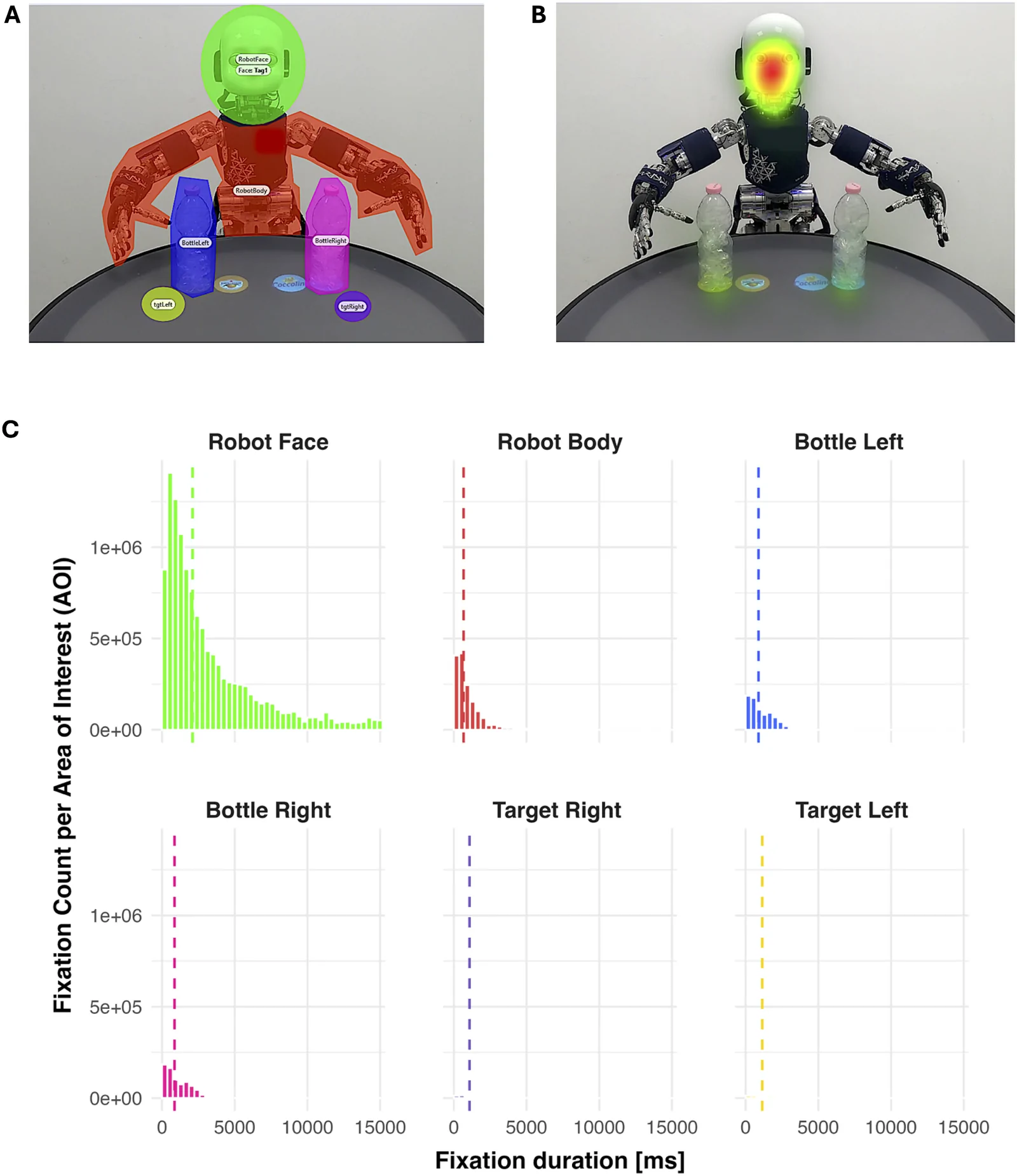
Fixations per area of interest (AOI)*.* AOIs included in the analysis segmented relevant areas in the interaction (A). A heatmap of fixations (above 60 ms) across all participants revealed that participants fixated on the robot's face (B). Faceted histograms per AOI display fixation durations in milliseconds, dashed vertical lines mark AOI-specific medians. All panels share the same x-axis scale (0 to the 99th percentile) to enable direct comparison (C). Participants fixated the robot's face for the majority of the recording, consistent with the interpretation that reaction-time effects reflect covert rather than overt orienting.

### 3.4 Discussion Experiment 2

Experiment 2 tested whether gaze orienting was modulated by *Action Congruency* during a live handover task with a robot co-actor. Participants stood before iCub, heard a contextual cue (drink, laundry, neutral), observed the robot look toward either the goal-aligned or goal-misaligned bottle, and then identified a briefly presented target letter at a spatially valid or invalid location. We predicted a standard orienting benefit (faster valid than invalid responses) that would be selectively amplified when gaze fit the action context. The data matched these predictions: reaction times showed a main effect of *Validity* and, critically, an *Action Congruency* × *Validity* interaction, with robust orienting in action congruent and neutral contexts, with no reliable orienting in incongruent contexts. Accuracy also favored goal-aligned over neutral trials.

Eye-tracking data confirmed sustained fixation on the robot's face (78.84% of all fixations), validating that participants maintained central fixation as instructed. Trials were not excluded based on fixation, so occasional looks toward the bottles are included in the data. The predominance of face fixation indicates that the reaction-time effects were driven mainly by attentional shifts unaccompanied by eye movements to the cued location rather than by overt orienting. This shows that the observed gaze-cueing effects reflect covert attentional orienting, shifts of spatial attention without corresponding eye movements, consistent with classic gaze-cueing paradigms (Driver et al., 1999; Friesen & Kingstone, 1998).

Action congruency modulated reaction times without affecting overt fixation patterns, indicating that the gaze-weighting heuristic extracts directional information from the robot's gaze to guide covert attention. Thus, classic gaze-cueing effects extend from screens to second-person, embodied exchanges in goal-directed joint action settings (Kompatsiari et al., 2018; McKay et al., 2021; Perez-Osorio, Müller, Wiese and Wykowska, 2015, Perez-Osorio, Müller and Wykowska, 2017; Wiese et al., 2012; Marchesi et al., 2025), and they do so selectively, being deployed when gaze aligns with the unfolding action plan and not deployed when it conflicts with shared task goals (Adolphs, 2003; Gallagher & Frith, 2003).

This modulation by action context aligns with prior evidence that task- and goal-related expectations shape social orienting (Wiese et al., 2012; Wykowska et al., 2014) and converges with demonstrations that gaze orienting is amplified when gaze matches predicted action sequences and attenuated when it conflicts (Perez-Osorio, Müller, Wiese and Wykowska, 2015, Perez-Osorio, Müller and Wykowska, 2017, Perez-Osorio, Abubshait and Wykowska, 2021). It is also consistent with findings that gaze anticipates upcoming actions (Flanagan & Johansson, 2003; Hayhoe et al., 2003) and with accounts proposing a flexible “syntax” of action that supports prediction (Maffongelli et al., 2015, Maffongelli, D'Ausilio, Fadiga and Daum, 2019). From a joint action perspective, these findings reveal how partners use social cues flexibly during coordination: participants integrated the robot's gaze when it supported their understanding of shared goals (congruent context), but did not deploy the robot's gaze when it conflicted (incongruent context). This pattern demonstrates that the gaze-weighting heuristic is not simply triggered by directional social cues but is strategically deployed based on whether those cues are relevant to the joint task at hand.

Our results also raise the question of whether these effects reflect the influence of iCub's gaze or the attentional pull of the object made relevant by the action context. Two features of the data indicate that gaze contributes independently. In the neutral context, where no object was made relevant, responses were nevertheless faster at the gazed-at location (23 ms, *p* = .021), which a purely context-driven account cannot produce. Furthermore, comparing conditions in which the target appeared at the context-matching bottle, responses were 23 ms faster when iCub had also looked toward it than when it had looked elsewhere, whereas conditions in which the target appeared at the non-matching bottle did not differ statistically. Action context, therefore, cannot account for the pattern independently of gaze. This converges with Perez-Osorio et al. (2015), who tested this alternative directly by analyzing trials on which the agent's gaze remained straight ahead and found no reliable effect of target compatibility with the action context.

The eye-movement results further support the conclusion that these effects reflect genuine attentional mechanisms rather than overt oculomotor responses. Participants looked at the robot's face as instructed while covertly attending to task-relevant locations. This covert deployment of the heuristic allowed participants to extract directional information from the robot's gaze without disrupting their own task performance. Our findings converge with theories of triadic joint attention and coordination toward shared goals (Fiebich & Gallagher, 2013; Frischen et al., 2007; Sebanz & Knoblich, 2009; Tomasello & Carpenter, 2007) and with proposals that social expectations guide attention to intentional cues such as gaze (Baron-Cohen, 1997; Frith & Frith, 2006; Stephenson et al., 2021). Together with Experiment 1, these findings demonstrate that the gaze-weighting heuristic is calibrated by both partner characteristics (reliability, Experiment 1) and situational factors (goal congruency, Experiment 2), operates flexibly under varying task demands, and manifests primarily as covert attentional shifts, a pattern consistent with natural joint action where partners maintain eye contact while coordinating their attention and actions in shared task spaces.

## 4 General discussion

During joint action, partners continuously coordinate attention and actions to achieve shared goals (Sebanz et al., 2006; Vesper et al., 2010). A fundamental mechanism supporting this coordination is the ability to extract directional information from a partner's gaze. Decades of work show robust gaze-orienting effects, but the extent to which the use of a partner's gaze reflects automatic processing versus strategic, context-dependent deployment remains debated. We addressed this by embedding gaze in live human–robot interaction and probing the question at two points in the processing stream: whether a partner's gaze informs an ongoing decision, manipulating informative validity together with task difficulty and agent reliability (Experiment 1), and whether it orients attention, manipulating goal alignment together with spatial validity (Experiment 2).

**Experiment 1** tested whether people selectively deploy the gaze-weighting heuristic as a function of robot reliability (Random: 50% vs. Reliable: 80%) and task difficulty. Validity benefit emerged only when the robot was reliable and the task was difficult. This demonstrates that the heuristic is calibrated by partner reliability, a partner-level prior. The *cost of reliance* on the robot was evident: reliable group participants made more errors on high-difficulty invalid trials, precisely because they relied on gaze. These results extend joint action research by demonstrating that partner modeling mechanisms operating in human-human coordination (Vesper et al., 2017) apply to human-robot interaction, suggesting flexible, utility-weighted processes for managing coordination.

**Experiment 2** tested whether action congruency modulates gaze orienting during a handover task. Validity benefits emerged when gaze pointed to the action-congruent object and in the neutral context, but when gaze pointed to the action-incongruent object. From a joint action perspective, this reveals how partners use shared task representations to gate social attention mechanisms. The auditory cue established a shared action frame specifying which object was task-relevant. When the robot's gaze aligned with this frame, participants integrated the signal; when it conflicted, they did not deploy it. This shows that the brain evaluates social cues against internal models of shared goals, deploying attentional resources only when cues are task-relevant. Eye-tracking data confirmed predominat face fixation while covert attention shifted based on gaze, a dissociation demonstrating that the heuristic operates at the level of attentional resource allocation rather than reflexive oculomotor responses.

The canonical gaze orienting effect survived the translation from screen to ecologically valid interaction, but its magnitude scaled with gaze utility and top-down predictions. Benefits appeared when the reliance on the observed gaze increased under high-difficulty and when the robot had proven reliable. Similarly, gaze orienting effects were observed when the robot's gaze was congruent with the action plan and were reduced when it conflicted. Eye tracking corroborated these effects reflected covert attentional shifts, as participants maintained fixation on the robot's face (78.84% of fixations) while shifting attention based on gaze cues. These results show that in embodied joint action contexts, the use of partners' gaze operates as a flexible, utility-weighted heuristic tuned by expectations, goals, difficulty, and reliability, rather than a purely reflexive response. Gaze effects were systematically modulated by reliability and task demands at the level of decision making, and by goal congruency at the level of attentional orienting. Together this indicates a mechanism that weights expected utility rather than responding reflexively to directional social stimuli.

A key contribution of our studies is replicating and extending screen-based results in a controlled yet lifelike laboratory interaction with a humanoid partner. Importantly, the interactive nature of our tasks meant that participants were not mere observers but active co-actors whose performance depended on successfully integrating their own perceptual judgments with the robot's social signals, an indicator of joint action coordination (Knoblich et al., 2011). The pattern aligns with studies showing that humans treat robotic gaze as socially meaningful; and that top-down factors, such as perceived social relevance or interactional framing, modulate sensitivity to robot signals (Abubshait et al., 2020; Abubshait & Wiese, 2017; Dalmaso et al., 2020; Gobel et al., 2018; Kompatsiari, Tikhanoff, Ciardo, Metta and Wykowska, 2017, Kompatsiari, Pérez-Osorio, De Tommaso, Metta and Wykowska, 2018, Kompatsiari, Ciardo, Tikhanoff, Metta and Wykowska, 2019; Perez-Osorio et al., 2021; Pérez-Osorio et al., 2018; Wiese et al., 2012; Wiese, Shaw, et al., 2017; Wiese, Weis, & Lofaro, 2018). Embedding gaze within goal-directed exchanges provides an ecological valid route for studying joint action and joint attention mechanisms (Wykowska, 2021; Wykowska et al., 2016).

We would like to emphasize that iCub's directional signal comprised a coordinated head-and-gaze shift, so eye direction and head orientation contributed jointly to the cue. This reflects a broader feature of orienting outside screen-based paradigms. Where classic gaze cueing isolates a discrete shift of the eyes, orienting in dynamic, ecologically valid settings is inherently composite, involving eyes, head and posture moving together toward a common target. The signal our participants used was therefore composite rather than gaze-specific, and we take this to be a property of the phenomenon in real interaction rather than an artifact of our implementation. Isolating the contribution of eye direction alone would require decoupling it from head orientation, which would produce a less representative cue, and is better suited to studies whose aim is mechanistic decomposition rather than ecological validity.

### 4.1 The gaze-weighting heuristic during joint action

Our findings support a theoretical account in which gaze-weighting during joint action functions as a predictive heuristic that guides how a partner's gaze is used, both in forming decisions and in allocating attention. Rather than automatically triggering a response, observed gaze serves as evidence weighted according to its expected utility for the current joint task. This weighting integrates the partner's reliability history (Experiment 1), the alignment between gaze and shared action goals (Experiment 2), and current task demands (difficulty). When these factors converge, when a reliable partner's gaze aligns with shared goals during a challenging task, the heuristic is deployed and performance benefits emerge. When they diverge, the heuristic is not deployed and gaze has minimal influence.

This positions gaze-weighting within predictive processing frameworks for social interaction (Friston & Frith, 2015; Kilner et al., 2007). During joint action, the brain generates predictions about a partner's intentions based on prior interactions and current context (Sebanz & Knoblich, 2009; Vesper et al., 2017). A partner's gaze provides evidence that either confirms or violates these predictions. Our findings suggest participants upweighted signals from reliable sources in appropriate contexts and down-weighted them otherwise, effectively “turning off” when the expected utility was low.

### 4.2 From reflexes to predictions: a tuned heuristic for social attention

Across both experiments, the gaze-weighting heuristic during joint action operates as a second-person control policy modulated by three filters: *action congruency* (does gaze belong to the current action grammar?), *task demand* (is help needed?), and *reliability* (has this partner been right?). This triad provides the calibration needed for coordinated joint action. The theoretical implications support a shift from reflexive processes to prediction and utility. That is, gaze is integrated into an online model of the task and the partner, and its weight is adjusted accordingly. Importantly, this also occurred without overt attentional shifts, with participants extracting directional information while largely maintaining fixation on the face across conditions. This allowed flexible resource allocation while preserving the social signal of sustained eye contact, consistent with task instructions. Note that in natural actions, people tend to look more toward the objects they need to manipulate. It is plausible to assume that the observed effects would be amplified by overt attentional shifts.

These findings make three contributions to joint action research. First, mechanisms supporting joint action (shared task representations, partner modeling, predictive coordination) also operate when the co-actor is robotic, suggesting flexible, utility-weighted processes triggered by behavioral cues rather than conspecific-specific systems. Second, our work extends joint task representations (Sebanz et al., 2006) by showing how high-level representations of shared goals gate lower-level perceptual mechanisms, suggesting a hierarchical architecture in which shared goals modulate social signal processing. Third, the covert nature of orienting highlights that partners must balance multiple attentional demands, maintaining eye contact for ongoing social monitoring and in line with task instructions while covertly attending to workspace objects, a fundamental feature enabling seamless coordination.

### 4.3 Limitations and future directions

Our design cannot determine whether an incongruent action context prevents gaze-driven orienting from occurring or whether orienting occurs and is subsequently offset by attentional allocation toward the context-relevant object. Both possibilities are consistent with the gaze-weighting account, since on either account, the net influence of a partner's gaze is set by the action context. Discriminating between them would require decoupling gaze direction from object identity, or temporally sensitive measures capable of detecting orienting that is initiated and then not deployed.

Additionally, in our study, reliability effects were evident in accuracy. Future work might employ within-participants designs where reliability drifts over time, allowing trial-by-trial belief updates. Furthermore, an open question remains regarding the processing stages at which difficulty and reliability exert their influence. There are three possibilities: (1) early sensory weighting (e.g., N1 ERP of the EEG signal, posterior alpha band), (2) control/conflict monitoring (midfrontal N2 ERP/theta band), and (3) decision accumulation models. EEG/pupillometry with hierarchical diffusion modeling could reveal whether the heuristic modulates early perceptual processing, decision-making, or both, specifying how the brain implements predictive resource allocation during joint action. To maintain control, embodiment was restricted (scripted gaze, limited head motion, no speech). Future work could test the effects in less controlled and even more natural interactions.

### 4.4 Gaze-weighting in human-robot interaction

Humanoid robots provide controlled yet embodied tests of joint action mechanisms. Our findings align with evidence that robot gaze elicits human-like attentional responses, and we add that people use those signals strategically when they fit the task structure and come from reliable partners. For human-robot collaboration, three design principles follow: (a) align gaze with actual action plans, (b) establish and maintain reliability or provide confidence signals, and (c) amplify guidance under high difficulty. Critically, miscalibrated gaze does not simply reduce effectiveness, rather it inverts the communicative function, transforming predictive cues into noise. Unreliable or incongruent gaze signals may delay joint action and degrade trust over time, defining a clear operational boundary in which gaze must remain dynamically coupled to both the robot's internal goals and the user's interpretation of task context and requirements.

## 5 Conclusion

By embedding gaze into live, goal-directed human-robot joint action, this work shows that classic gaze-cueing effects persist in interactive scenarios but are systematically modulated by predictive beliefs about action goals and experience-based reliability estimates. Rather than operating as an automatic process, the use of a partner's gaze during joint action functions as a strategic heuristic guiding both decisions and attentional allocation according to expected utility for joint task performance. This heuristic allows partners to extract directional information demonstrating multi-level flexibility required for natural joint action. Our findings advance social-attention theory by demonstrating that the use of a partner's gaze is better understood as a predictive, context-dependent mechanism for resource allocation during joint action, and offer practical principles for designing gaze-aware robots that support seamless human–robot collaboration.

## CRediT authorship contribution statement

**J. Perez-Osorio:** Writing – review & editing, Writing – original draft, Visualization, Validation, Resources, Methodology, Investigation, Formal analysis, Data curation, Conceptualization. **E. Wiese:** Writing – review & editing, Writing – original draft, Methodology. **A. Abubshait:** Writing – review & editing, Writing – original draft, Validation, Methodology, Investigation, Formal analysis, Data curation, Conceptualization. **A. Wykowska:** Writing – review & editing, Writing – original draft, Supervision, Resources, Project administration, Funding acquisition, Conceptualization.

## Declaration of Generative AI and AI-assisted technologies in the writing process

During the preparation of this work the corresponding author used Claude (Anthropic) in order to support manuscript editing, including improving clarity and readability, and checking consistency between in-text citations and the reference list. After using this tool, the authors reviewed and edited the content as needed and take full responsibility for the content of the published article.

## Funding

This work has received support from the 10.13039/501100000781European Research Council under the European Union's Horizon 2020 research and innovation program (Grant ERC-2016-StG-715058) awarded to Agnieszka Wykowska and internal funding from the 10.13039/501100009531Istituto Italiano di Tecnologia, Genova. The content of this article is the sole responsibility of the authors. The European Commission or its services cannot be held responsible for any use that may be made of the information it contains.

## Declaration of competing interest

The authors declare that they have no known competing financial interests or personal relationships that could have appeared to influence the work reported in this paper.

## Data Availability

I have provided a link to the data in the attach file step.
