# Supplementary material for: Predictions and goals modulate use of partner's gaze during joint action with humanoid robots
Source: Acta Psychol (Amst). 2026 Oct;270:107838. doi: 10.1016/j.actpsy.2026.107838 (PMC13724303; doi:10.1016/j.actpsy.2026.107838)
Supplement: Supplementary file 1 [file mmc1.docx]

**SUPPLEMENTARY DATA**

A. **Reaction times analysis**

The 2 × 2 × 2 mixed ANOVA conducted on reaction times revealed a significant main effect of *Difficulty* (*F*(1, 47) = 10.09, *p* = .003, partial η² = .18), with participants responding more slowly on trials with high-difficulty compared to low-difficulty trials. No main effects of *Reliability* (*F*(1, 47) = 0.002, *p* = .968) or *Validity* (*F*(1, 47) = 0.38, *p* = .540) were observed. The *Validity* × *Difficulty* interaction was significant (*F*(1, 47) = 4.92, *p* = .031, partial η² = .10), indicating that the validity effect varied by task difficulty. The three-way interaction between *Reliability,* *Validity*, and *Difficulty* was not significant (*F*(1, 47) = 0.58, *p* = .450); see **Fig. S1**.

**Fig. S1**. *Reaction times by reliability, validity, and difficulty.* Responses slowed under high difficulty, particularly on invalid trials. Reliable and random groups showed similar overall speeds, though slowing was more pronounced for the reliable group. Error bars represent the Standard Error.

Simple contrast effects tests following the significant Validity × Difficulty interaction showed that the validity effect was present only under high difficulty: invalid trials were on average 19 ms slower than valid trials, *t*(47) = 2.32, *p* = .025 (Δ = –.019 s, SE = .008). Under low difficulty, the validity difference (9 ms) was not significant, *t*(47) = 0.71, *p* = .48. Conversely, difficulty slowed performance only on invalid trials. For valid trials, the high- minus low-difficulty difference was negligible (5 ms), *t*(47) = 0.93, *p* = .36, whereas invalid trials showed a significant 33 ms cost of high difficulty, *t*(47) = 3.00, *p* = .004. Because the Reliability × Difficulty effect approached significance, difficulty was also probed within each group, **see Fig. S1**. The high-difficulty cost reached significance for reliable (31 ms), *t*(47) = 3.60, *p* = .001, but was nonsignificant for random (7 ms), *t*(47) = 0.86, *p* = .39.

Taken together, difficulty consistently affected reaction times, and the validity effect depended on difficulty, but no robust group differences or three-way interactions were observed. High task difficulty selectively lengthened reaction times on invalid trials, an effect driven primarily by participants in the reliable group. Validity differences emerged only under the most demanding condition.

B. **Apparatus Experiment 1**

The robot was connected to a single workstation (SSD 250 Gb, 32 Gb RAM, Intel i7 7700CPU@3.60 GHz 4 cores, GPU GeForce GTX 1060 3Gb, running Ubuntu 20.04 LTS) through a peer-to-peer Ethernet connection. iCub’s behavior and the stimuli presented on the screen were controlled with Psychopy 2020.1.2 (Peirce et al., 2019). iCub’s movements and gaze behaviors were pre-programmed joint positions to facilitate the replication of the joint positions and minimize the variability across trials and participants. The robot's movements were controlled using YARP (Metta et al., 2006) and Python wrappers. The robot gaze behavior was implemented using the iKinGazeCtrl (6-DOF controller), with a neck trajectory time of 600 ms and an eye trajectory time of 250 ms executed simultaneously, so that the directional gaze cue was configured to complete in approximately 600 ms. Because iCub's movements are subject to mechanical jitter of approximately 100 ms, the exact duration varied slightly across trials. Each head/gaze shift toward a container was followed by a torso rotation toward the same side, with the whole movement settling within approximately 1200 ms. Participants used cubes of 8.5 cm made of polystyrene foam and covered with colored duct tape. The cubes were light and easy to sanitize after every participant’s use.

C. **Apparatus Experiment 2**

OpenSesame and the iCub middleware YARP were used to control the robot's movements. A processing unit (SSD 250 Gb, 32 Gb RAM, Intel i7 7700CPU@3.60 GHz 4 cores, GPU GeForce GTX 1060 3Gb, running Ubuntu 12.04 LTS) used to connect (a) the iCub robot through a peer-to-peer Ethernet connection, (b) the table projector through HDMI and (c) the eye-tracker glasses through a Wi-Fi connection. All software modules in the experiment were integrated into a single workstation. To meet the experiment's requirements, iCub performed two tasks: (a) examining an object placed on the table (task 1), and grasping one of these objects to hand it to the participant (task 2). For ***task 1***, we implemented a Python wrapper of a 6-DOF gaze controller, which can track a 3D Cartesian fixation point, calculating neck and eye poses to look at that point. We configured four different 3D target points: (a) the object on the left, (b) the object on the right, (c) the participant’s face, and (d) the center of the table. iCub moved both its eyes and its neck to indicate one of the bottles. Eyes and the neck of iCub were controlled by the YARP Gaze Interface, *iKinGazeCtrl*. The vergence of iCub’s eyes was set to 2 degrees, and the tilt was maintained at +/- 3 degrees during the experiment. To ensure the same robot movement for all the conditions, the y-coordinates of “left” and “right” were the same.

The trajectory time for eye and neck movement was set to 800 ms and 1200 ms, respectively, to maintain the impression of smooth, naturalistic movement. Eyes and neck moved simultaneously. For ***task 2***, we implemented a Python wrapper of an iCub module called Constant Time Position Service. This service allows sending target joint positions and the trajectory time for executing the movement. Then, the velocity profiles and the joint trajectories are generated to reach that target position within the required time. All the robot movements for manipulating the objects were pre-recorded to ensure minimal variability in terms of time and joint positions.
